# Serum Albumin Concentrations in a Multi-Ethnic Cohort of Patients with Human Immunodeficiency Virus Infection from South East London

**DOI:** 10.1089/biores.2014.0038

**Published:** 2015-02-01

**Authors:** James JY Chong, Ellen Fragaszy, Oliver Dukes, John Cason, Zisis Kozlakidis

**Affiliations:** ^1^ King's College London, School of Medicine, Guy's Campus, Guy's Hospital, Great Maze Pond, London, United Kingdom.; ^2^ University College London, Institute of Health Informatics and the Farr Institute of Health Informatics Research, London, United Kingdom.; ^3^ University College London, Infection and Immunity and the Farr Institute of Health Informatics Research, London, United Kingdom.

**Keywords:** virology, HIV, biomarkers

## Abstract

Human albumin is the most abundant protein in sera and a valuable biomarker in monitoring a variety of diseases. In this study we investigated the relationship between serum albumin concentrations and effects of initiation of highly active antiretroviral therapy (HAART). Serum albumin concentrations amongst 70 HIV-infected patients from diverse ethnicities were analyzed, in the absence of any other confounding comorbidities, over a period of 8 years in South East London, United Kingdom. Serum albumin data was collected, on average, every 4–6 weeks during routine visits. Serum albumin was measured prior to starting HAART, and measured at the first clinic visit after commencing HAART. These were compared to a control group of untreated individuals. Based on our analyses we conclude that serum albumin concentrations increase significantly after the initiation of therapy.

## Introduction

A link between circulating albumin levels, infection, and mortality has been documented,^1,2^ with serum albumin concentrations decreasing significantly during periods of stress, trauma, or sepsis.^3^ Albumin can also be lost into the intestine, where digestion releases amino acids and peptides that can be reabsorbed in healthy subjects.^4^ In an intensive care setting, mortality and infection rates increased in critically ill patients with hypoalbuminaemia (below 35g/L).^1^ Serum albumin has been recognized as a prognostic indicator in human immunodeficiency virus infection (HIV).^5–7^ Graham et al. (2007) reported on the association between serum albumin and HIV in a cohort of HIV-positive women in Kenya: each 1g/L decrease in serum albumin was associated with a 13% increase in risk of progression to a CD4 T-cell count of <200 cells/mm^3^.^8^

There is just one report on the effect of highly active antiretroviral therapy (HAART) on serum albumin concentrations amongst those infected with HIV. Chauhan et al. 2011 studied HIV-positive Indian patients and found that serum albumin levels increased after the commencement of HAART.^9^ In the long term, HAART has the potential to cause hepatotoxicity and may impair albumin production.^10^

Our study investigated serum albumin concentrations in a cohort of HIV-positive patients in South East London, United Kingdom. We investigated whether the initiation of HAART impacts upon serum albumin concentrations, compared to a control group of untreated HIV-positive individuals.

## Materials and Methods

Seventy HIV-1 positive patients recruited to the Infectious Diseases BioBank (IDB)^11^ at King's College London were investigated. Thirty-nine of these patients commenced HAART, and baseline serum albumin levels immediately prior to treatment and the first measurement after treatment were used, generally within 4–6 weeks. These were analyzed against the control group of 31 untreated patients, where the baseline serum albumin level was the earliest recorded measurement. The median measurement from multiple clinic visits was taken in the control group to compare to post-HAART serum albumin in the treated group. Of the 70 patients, none were known to be hepatitis B virus positive. Data between July 2004 and June 2013 was included, and on average, serum albumin concentrations were measured every 4–6 weeks for each patient. Normal serum albumin concentrations were considered to be between 35 and 50g/L.^12^

Baseline CD4 T-cell count data was measured in the treated cohort immediately prior to treatment, and measured again after treatment, generally within 4–6 weeks. These were analyzed against the untreated control group, where the baseline CD4 T-cell count was the earliest recorded measurement. The median measurement from multiple clinic visits was taken in the control group to compare to post-HAART CD4 T-cell count in the treated group. Serum albumin concentrations and CD4 T-cell counts were measured by GSTS Pathology. Patients infected with a variety of HIV clade strains were represented in both cohorts.

Ethical approval for all aspects of this study was obtained through the King's College London Infectious Disease BioBank Local Research Ethics Committee (under the authority of the Southampton and South West Hampshire Research Ethics Committee—approval REC09/H0504/39). Statistical testing was done by comparing the mean change from baseline level of serum albumin/CD4 T-cell count to post-HAART or median measurement between treatment groups, using a t test with change scores, and statistical analyses were performed using Stata 13.1.

## Results

Of the 70 patients, the majority were men (85.7%) and white (67.1%), followed by black Africans (14.3%) and other black (10%). The mean age at diagnosis was 33.7 (±8.4) years. Patient characteristics are listed in Table 1.

**Table 1.** **Patient Characteristics**

	*Untreated (*n*=31)*	*Treated (*n*=39)*
Mean age at diagnosis, years (SD)	31.5 (±6.5)	35.5 (±9.3)
Mean age at commencement of HAART, years (SD)	N/A	38.6 (±9.6)
Mean CD4 T-cell count, baseline, cells/mm^3^ (SD)	646.2 (±255.3)	299.5 (±124.1)
Mean CD4 T-cell count, median/after treatment, cells/mm^3^ (SD)	664.6 (±293.9)	470.3 (±201.8)
Sex, *n* (%)		
Male	24 (77.4%)	36 (92.3%)
Female	7 (22.6%)	3 (7.7%)
Ethnicity, *n* (%)		
White	17 (54.8%)	30 (76.9%)
Asian	0	1 (2.5%)
Black, African	7 (22.6%)	3 (7.7%)
Black, other	6 (19.4%)	1 (2.5%)
Mixed/other	0	4 (10.4%)
N/A	1 (3.2%)	0

SD, standard deviation; HAART, highly active antiretroviral therapy.

There was sufficient data to compare initiation of HAART for 39 patients. The majority of patients were male (92.3%) and white (76.9%). The mean age at diagnosis was 35.5 (±9.3) years, and the mean age at commencement of HAART in the 39 patients was 38.6 (±9.6) years.

**FIG. 1.**
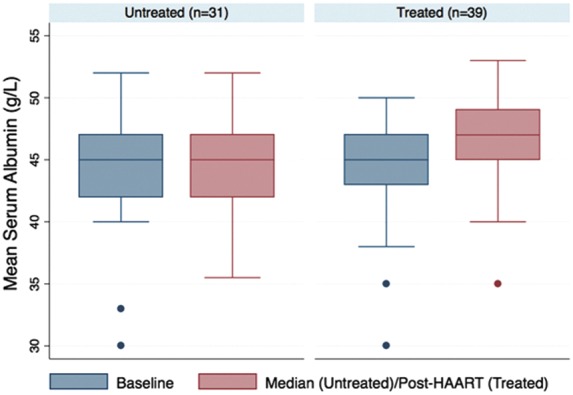
Mean serum albumin concentrations. HAART, highly active antiretroviral therapy.

**FIG. 2.**
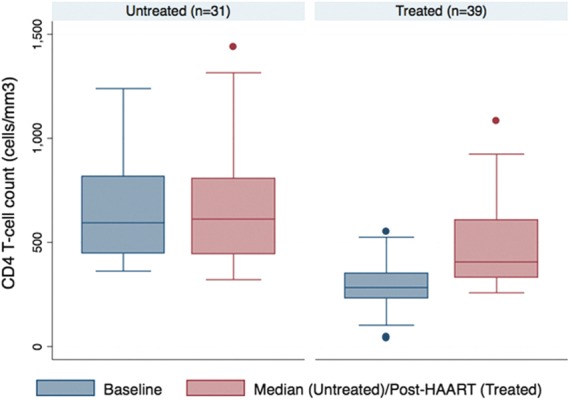
CD4 T-cell count, between baseline and median/after therapy.

The mean change in serum albumin between baseline and median in the untreated group was 0.24g/L (95% confidence interval [95% CI] −0.88 to 1.36), compared with pre- and post-treatment change of 2.15 g/L (95% CI 1.06 to 3.25) in the treated group. The mean change was 1.91g/L (95% CI 0.36 to 3.47) in the treated group compared with the untreated group. There was strong evidence that this change between the two time points differed between the treated and untreated groups (*p*=0.0166) (Fig. 1). The mean change in CD4 T-cell count between baseline and median in the untreated group was 2.16 cells/mm^3^ (95% CI −83.01 to 87.24), compared to pre- and post-treatment change in the treated group of 191.16 cells/mm^3^ (95% CI 126.57 to 255.75). Strong evidence suggests that this change between the two time points differed between the treated and untreated groups (*p*=0.0005) (Fig. 2).

## Discussion

Our data shows that serum albumin levels increase significantly after commencement of HAART, compared with a control cohort of untreated individuals. In comparison with the closest equivalent study, our patients were more ethnically diverse and presented with no hypoalbuminaemia at the baseline visit.^9^ The difference in baseline hypoalbuminaemia between our cohort and the Indian cohort may be due to dietary, geographic, and/or ethnic group differences. Additionally, there is little information on the HIV-1 clade in the other comparable studies to be able to investigate any potential correlation.

HAART has a profound impact on HIV, lowering viral loads and allowing CD4 T-cells to increase in number.^13^ Our data shows a significant increase in CD4 T-cell count after the initiation of HAART. In patients coinfected with other viruses, the beneficial effect of HAART on the immune system can result in lowering of viral loads in chronic infections such as hepatitis B^14^, C^15^, and G^16^. Despite the number of studies looking into coinfected individuals, most patients are likely to be HIV positive and hepatitis B/C negative, and the effect of HAART on liver function in monoinfected patients is of greatest clinical relevance.

Serum albumin levels are a good prognostic marker in HIV infection, with studies from both the pre-HAART^6^ and post-HAART^7^ eras supporting their relevance in HIV medicine. Mehta et al. (2006) reported that hypoalbuminaemia after HIV seroconversion was associated with faster disease progression, and a likely consequence of HIV infection.^17^ Additionally, in resource-poor settings, the potential use of serum albumin levels as an alternative to the more costly and specialized CD4 T-cell counts and viral load assays has been recognized.^18,19^ However, serum albumin levels are unlikely to respond as acutely as CD4 T-cell count and viral load assays, and where available, these should always be used as first line assays.

We hypothesize that in our control group of untreated individuals, the stress of their HIV infection may affect the rate of production for some proteins such as albumin, or increase their rate of loss. The observed differences in albumin levels may be due to multiple factors. Some of the possible explanations for the change in serum albumin levels include: (1) the fact that it is a “negative” acute phase protein and levels fall during inflammation, (2) excessive excretion via the kidney, (3) loss to the bowel, and (4) liver disease. The latter is unlikely as we excluded patients with known hepatitis B or C virus infections. Integrity of the bowel wall is damaged during the acute phase of HIV infection resulting in bacterial translocation into the plasma, and HIV infection causes inflammation of the bowel. The paradox in both of these cases is that such damage is believed to occur early during the disease process, yet we noted that albumin concentrations were lowest amongs those with chronic disease.

The presented data supports an observed benefit of HAART on circulating serum albumin levels and CD4 T-cell count. The relationship between HAART and the liver, the site of albumin synthesis, is a balance of the beneficiary effect of immune system reconstitution versus the potential long-term toxic nature of the antiviral drugs that constitute HAART.^20^ Although an increase of 2.15g/L may seem clinically small, this rise in serum albumin level is a likely indicator of the overall benefit of HAART in mitigating the systemic effects of HIV infection, as described above. In conclusion, commencement of HAART in HIV infected individuals exhibits benefits to the circulating serum albumin levels.

## Abbreviations Used

HAART: highly active antiretroviral therapy
HIV: human immunodeficiency virus
IDB: Infectious Diseases BioBank
